# Reply to comment of “ERK and p38MAPK combine to improve survival in patients with BRAF mutant colorectal cancer”

**DOI:** 10.1038/s41416-018-0275-7

**Published:** 2018-10-09

**Authors:** Antonia K. Roseweir, Donald C. McMillan, Joanne Edwards

**Affiliations:** 1 School of Medicine, University of Glasgow, Glasgow, UK; 2 Institute of Cancer Sciences, University of Glasgow, Glasgow, UK

**Keywords:** Tumour biomarkers, Cancer microenvironment

Sir,

We are grateful for the comments of Uzunparmak and Sahin regarding our paper on ERK and p38MAPK as prognostic markers for patients with BRAF mutations.^1^

It is well-recognised CpG Island methylator phenotype (CIMP) is a major cause of microsatellite instability in colorectal cancer due to methylation of MLH-1 and that this is frequently coupled with BRAF mutations. Unfortunately, we do not have CIMP analysis in the present study cohort and therefore are unable to comment on how this affects both Microsatallite instability (MSI) (measured using mismatch repair deficiency (dMMR) as a surrogate in this paper) and BRAF mutations within this cohort.

The authors suggest that the level of dMMR with BRAF mutations at 16% in our cohort was low compared with other studies and that in patients with sporadic dMMR, MLH-1 methylation or expression loss account for approximately 60% of patients that harbour BRAF mutations.^2^ However, in the present study dMMR was examined across the four common MMR proteins (MLH-1, PMS-2, MSH-2 and MSH-6) and not just MLH-1. If only MLH-1 was examined, then approximately 30% of patients had BRAF mutations. In addition, the present cohort was not tested for Lynch syndrome since there was no available germline DNA. Lynch syndrome although associated with dMMR it is not usually associated with BRAF mutation. Therefore, if some of the present cohort had Lynch syndrome this may have also diluted out the proportion of sporadic dMMR patients that had BRAF mutations. Irrespective, we will look to better characterise the present cohort in the future.

Although the pERK/p38MAPK score appeared to be most effective when restricted to stage III MMR competent patients, when all BRAF mutant patients were considered the pERK/p38MAPK score was still associated with poorer survival independent of TNM-stage suggesting a wider applicability in patients.

Clearly, the present work is required be confirmed in other cohorts, as external validation is key to any prognostic score being taken forward. However, the simplicity of the present approach is that in BRAF mutant patients it builds on MMR testing and stratifies the patients according to recognised signal transduction pathways.
